# The Role of Endoplasmic Reticulum Stress and Unfolded Protein Response in Atherosclerosis

**DOI:** 10.3390/ijms17020193

**Published:** 2016-02-01

**Authors:** Ekaterina A. Ivanova, Alexander N. Orekhov

**Affiliations:** 1 Department of Development and Regeneration, KU Leuven, Leuven 3000, Belgium; 2 Laboratory of Angiopathology, Institute of General Pathology and Pathophysiology, Russian Academy of Sciences, Moscow 125315, Russia; a.h.opexob@gmail.com; 3 Institute for Atherosclerosis Research, Skolkovo Innovative Center, Moscow 121609, Russia

**Keywords:** atherosclerosis, endoplasmic reticulum stress, ER stress, atherosclerotic plaque, complicated plaque, ER stress modulators

## Abstract

Pathogenesis of atherosclerosis is a complex process involving several metabolic and signalling pathways. Accumulating evidence demonstrates that endoplasmic reticulum stress and associated apoptosis can be induced in the pathological conditions of atherosclerotic lesions and contribute to the disease progression. Notably, they may play a role in the development of vulnerable plaques that induce thrombosis and are therefore especially dangerous. Endoplasmic reticulum stress response is regulated by several signaling mechanisms that involve protein kinases and transcription factors. Some of these molecules can be regarded as potential therapeutic targets to improve treatment of atherosclerosis. In this review we will discuss the role of endoplasmic reticulum stress and apoptosis in atherosclerosis development in different cell types and summarize the current knowledge on potential therapeutic agents targeting molecules regulating these pathways and their possible use for anti-atherosclerotic therapy.

## 1. Introduction

Atherosclerosis is one of the most challenging problems of current medicine, being the primary cause of the development of cardiovascular diseases that account for the majority of fatal illnesses in developed countries [1]. The development of atherosclerosis is a complex process, for which several risk factors have been established, including genetic predisposition, metabolic disorders, such as elevated plasma cholesterol and diabetes, and environmental factors, such as diet, exposure to tobacco smoke and insufficient physical activity [2]. Atherosclerotic lesion development is caused by lipid accumulation in the arterial wall accompanied by a significant increase of the wall thickness and local inflammatory process. Lesion development is apparently triggered by local endothelial dysfunction and increased permeability of the endothelium for the circulating lipoprotein particles. The main source of cholesterol deposit in the arterial wall is the atherogenic fraction of low-density lipoprotein (LDL). Small dense LDL particles that have been modified by oxidation, desialylation and glycation, and especially LDL complexes, are the main contributors to the pathological process, and their presence in circulation is therefore a major risk factor of atherosclerosis [3]. The development of atherosclerotic lesion is associated with local inflammation and the recruitment of circulating immune cells. Both resident arterial wall cells (such as pericytes and vascular smooth muscle cells) and infiltrated cells (such as macrophages) actively participate in the pathological process. Careful studies of young adults have demonstrated that atherosclerotic process can begin early in life and remain asymptomatic for a long period [4,5]. At later stages, atherosclerotic lesion can convert into so-called vulnerable plaque, which can initiate thrombosis leading to life-threatening events that can be the first clinical manifestations of the disease in many patients. The improvement of anti-atherosclerotic therapy should therefore be focused on early diagnostic and preventive treatment. Such improvement necessitates a better understanding of the molecular and cellular mechanisms of the disease pathogenesis [6].

There is accumulating evidence of the important role of the endoplasmic reticulum (ER) stress at all stages of the atherosclerosis initiation and progression [7]. The ER is a complex membrane compartment of the eukaryotic cell, which is responsible for proper maturation and folding of intracellular and secreted proteins. The ER has an important signalling function being the major cellular reservoir of calcium, which is actively pumped in from the cytoplasm and released back in response to certain stimuli. Normal concentration of calcium in the ER is essential for maintaining its functions. The protein folding capacity of the ER can be saturated in various conditions, including ischemia, oxidative stress, disturbances of calcium homeostasis, accumulation of folding-defective proteins or enhanced expression of normal proteins [8]. These conditions can therefore result in the development of ER stress.

## 2. Molecular Mechanisms of the Unfolded Protein Response

To alleviate the disturbance of ER homeostasis in stress situations, cells initiate the unfolded protein response (UPR). The three known mechanisms that trigger the UPR in mammalian cells are activation of stress sensors situated on the ER membrane: protein kinase RNA-like ER kinase (PERK), inositol-requiring protein 1 (IRE1), and activating transcription factor 6 (ATF6) [9]. Under normal conditions, the ER stress sensors are maintained in inactive state through association with a chaperone protein, glucose-regulated protein 78 (GRP78 or BiP) [10]. The negative regulator dissociates when the aggregation of misfolded proteins or other stress events take place and therefore triggers the initiation of the UPR.

Several mechanisms of ER stress alleviation by UPR exist. First, protein load of the ER can be reduced by general inhibition of protein translation [11]. Second, UPR induction is associated with activation of a transcriptional network involving ATF6, ATF4 and X-box binding protein 1 (XBP1), which regulates the expression of genes that help to restore ER homeostasis. In particular, enhanced chaperone production promotes protein folding in the ER. Activated XBP1 controls the transcription of components of the ER-associated degradation (ERAD) process, which reduces the load of misfolded proteins via ubiquitin-dependent degradation by the proteasome [12]. Third, the ER compartment can be enlarged by stimulation of ER biogenesis [9]. The UPR is therefore an adaptive pathway, which is aimed at stress reduction. However, chronic unresolved ER stress or acute stress conditions lead to activation of apoptotic pathways and cell death [13]. Therefore, ER stress can be both protective and pro-apoptotic depending on the circumstances, such as the intensity of the stress stimuli or cellular physiological state. ER sensor proteins, such as IRE1 and PERK, are involved both in the adaptive and the pro-apoptotic UPR pathways.

IRE1 is the fundamental ER stress sensor, which is conserved from yeast to humans. Upon dissociation with GRP78, IRE1 is activated by dimerization and autophosphorylation. It regulates the specific splicing of mRNA encoding XBP1 resulting in translation of functionally active XBP1. Active XBP1 translocates to the nucleus where it stimulates transcription of the ER chaperones and other UPR-related proteins. At the same time, IRE1 can induce degradation of certain mRNAs through regulated IRE1-dependent decay (RIDD) contributing to the attenuation of the ER overload. In chronic ER stress, IRE1α can be involved in activation of pro-inflammatory and apoptotic pathways. It can associate with the adaptor protein tumor necrosis factor (TNF) and tumor necrosis factor receptor-associated factor 2 (TRAF2) [14]. The resulting complex then recruits mitogen-activated protein kinase (MAPK), apoptosis signal-regulating kinase (ASK) and caspase-12 [15,16]. The complex activates nuclear factor κB (NF-κB) signalling, which controls the expression of a number of pro-inflammatory genes, therefore providing a link between ER stress and inflammation [17].

Similar to IRE1α, PERK is activated through self-phosphorylation after dissociation from GRP78. Activated PERK blocks cellular protein synthesis by phosphorylation of the eukaryotic initiation factor 2α (eIF2α), which is necessary for the induction of cap-dependent transcription. This results in the reduction of the ER protein load [9]. At the same time, phosphorylated eIF2α regulates translation of certain mRNAs, including the mRNA of the transcriptional factor ATF4. This provides a negative feedback loop for the regulation of protein synthesis, since ATF4 activates expression of GADD34, a component of phosphatase responsible for dephosphorylation of eIF2α [18].

The ATF4 and, to a lesser extent, ATF6 and XBP1 factors regulate expression of CCAAT/enhancer-binding protein homologous protein (CHOP) [19]. CHOP is a leucine zipper transcription factor, which can induce apoptosis through several mechanisms. One such mechanism is up-regulation of ER oxidase 1 (ERO1)α and calcium-dependent apoptotic pathway. ERO1α up-regulation can lead to production of hydrogen peroxide, a by-product of disulfide bond formation, reactive oxygen species (ROS) overproduction and stimulation of oxidative stress [20]. Activation of ERO1α stimulates inositol-1,4,5-triphosphpate receptor-1 (IP3R1) [21], which leads to a depletion of the ER calcium store and increase of the cytoplasmic calcium concentration. The latter promotes stimulation of calcium/calmodulin-dependent protein kinase II, which is involved in several pro-apoptotic pathways [22]. Another important pro-apoptotic pathway of CHOP is regulation of the balance of pro- and anti-apoptotic B-cell lymphoma (Bcl)-2 family proteins. Under the ER stress conditions, CHOP suppresses the expression of anti-apoptotic Bcl-2 and Bnip3 and induces production and translocation to the ER membrane of pro-apoptotic Bim [23,24].

ATF6 is activated during the ER stress by cleavage and translocation of the cytosolic N-domain into the nucleus, where it stimulates expression of several UPR-related genes, including GRP78 and XBP1 [11]. ATF6 also induces expression of Derlin-3, which enhances the ERAD activity [25].

## 3. Role of ER Stress and Unfolded Protein Response (UPR) in Atherosclerosis

Atherosclerotic plaque provides conditions that can trigger ER stress and UPR, including inflammation, presence of oxidized lipids and metabolic changes [26]. In particular, oxidized LDL (oxLDL) was demonstrated to induce ER-stress and apoptosis in cultured cells of human arterial wall. Exposure of endothelial cells and macrophages to oxLDL led to inactivating modification of protein disulfide isomerase (PDI), an important ER chaperone responsible for disulfide bond formation. Overexpression of PDI had protective effects, and inhibition further enhanced oxLDL-induced apoptosis [27]. Incubation of endothelial cells with oxLDL also resulted in increased expression of the ER chaperone oxygen-regulated protein (ORP)150, which alleviated the oxLDL-induced apoptosis [28]. It is likely that inactivation of the protective system of ER chaperones, which is induced in arterial wall cells in response to toxic effects of modified LDL, may contribute to the increased cell death and complicated plaque formation in atherosclerosis. Indeed, increased levels of ORP150 and modified PDI were detected in advanced atherosclerotic lesions, suggesting that inactivation of anti-apoptotic chaperones can occur *in vivo* contributing to the disease progression.

Evidence of ER stress and UPR activation in atherosclerotic lesions comes from studies of marker proteins expression in human tissues [29]. Analysis of autopsy material demonstrated a strong association of the ER stress markers GRP78, GRP94 and CHOP in smooth muscular cells and macrophages from thin-cap atheroma and ruptured plaques in comparison with tissues at other stages of lesion development (initial intimal thickening, fibrous plaques and thick-cap atheroma) [29]. It is therefore likely that UPR activation is associated with advanced stages of atherosclerosis with serious clinical complications.

The role of the ER stress and UPR in atherosclerosis pathogenesis has been studied on animal models. In atherosclerotic mice, evidence of UPR activation could be observed at different stages of lesion development: in macrophages from areas of intimal thickening, fatty streaks and complex lesions [30]. Macrophages with and without lipid accumulation, as well as smooth muscle cells from atherosclerotic lesions had increased expression of the ER stress markers PDI, GRP78 and calreticulin. XBP1 protein in both active and inactive forms was increased in the advanced lesions and phosphorylated PERK was present, especially in the cap area. Another marker induced by ER stress, T cell death-associated gene 51 (TDAG51) [31], was found in atherosclerotic lesions at all stages. High levels of CHOP mRNA were detected in macrophage-rich advanced atherosclerotic plaques in mice. Moreover, the experiments conducted in mice demonstrated a direct causal link between ER stress-induced apoptosis and plaque necrosis, as the latter was reduced when ApoE or LDL receptor knock out atherosclerotic mice were bred with CHOP-deficient mutant animals [32]. Together, these observations indicate that ER stress and UPR are induced in atherosclerosis and play a significant role in the disease pathogenesis.

## 4. Endoplasmic Reticulum (ER) Stress in Different Cell Types of the Arterial Wall

Atherosclerotic lesion begins with the local disturbance of endothelial function [33]. Endothelial cells are subject to the continuous tension of the blood flow, and certain areas of the vessels where laminar flow is disturbed, for instance, by vessel bending or branching, are especially vulnerable. Analysis of transcript profiles from arterial regions susceptible or resistant to atherosclerosis isolated from non-atherosclerotic pigs revealed the presence of ER stress markers in atherosclerosis-susceptible areas [34]. In these regions, IRE1, ATF6 and XBP1, but not PERK and eIF2α were activated, indicative of the increased chaperone production. ATF4 and CHOP were increased at mRNA, but not protein level. These results suggest that UPR activation at early stages of atherosclerosis plays a protective role in response to stress induced by disturbed blood flow. ER stress, according to a GRP78 marker, was markedly increased in endothelial cells subjected to shear stress [35]. ER stress with activated IRE1 and CHOP pathways can also be induced in endothelial cells by oxidized phospholipids and homocysteine, the well-known atherosclerosis inducers [36,37,38]. Study on a rabbit model of atherosclerosis demonstrated that CHOP expression was associated with endothelial cells apoptosis at more advanced stages of the plaque development [39]. Exposure to modified (oxidized and glycated) LDL led to the development of oxidative stress and disturbed calcium signalling in the ER [27,28,40], contributing to the ER stress-mediated endothelial dysfunction and atherogenesis. In summary, ER stress induced in endothelial cells as a result of physical factors or exposure to pro-atherosclerotic molecules, such as modified LDL, can fail to exert its protective functions and instead lead to apoptosis and endothelial dysfunction, which plays a crucial role at the early stages of lesion development. At the same time, ER stress-induced apoptosis may be directly implicated in the thrombosis development at late stages of atherosclerosis [41].

In macrophages, engulfed LDL particles can be transported from the endosomal system to the ER, where cholesterol is esterified and stored in a form of inert lipid droplets [42]. In atherosclerosis, lipid metabolism in macrophages is altered, and ER-mediated cholesterol esterification fails to prevent the intracellular accumulation of non-esterified cholesterol [43]. The resulting foam cells, with cytoplasm filled with lipid droplets, contribute to the atherosclerotic plaque growth. In such cells, oxidoreductases present in the ER oxidize cholesterol to 7-ketocholesterol and other oxysterols that are highly toxic and can induce ROS-mediated damage and cell death [44]. Macrophage apoptosis induced by prolonged ER stress is a well-studied feature of advanced stages of atherosclerosis. Apoptosis associated with an enhanced expression of CHOP was demonstrated in atherosclerotic lesions in humans [31] and ApoE-deficient mice [32]. On the other hand, inactivation of CHOP had a protective effect in ApoE-deficient atherosclerosis mouse model reducing plaque necrosis [32,45]. Increased cell death and deficient clearance of the dying cells in advanced plaques are responsible for the formation of the inflammatory necrotic core [46]. It is likely that macrophage apoptosis *in vivo* is triggered not only by ER stress, but also by some “second hit” stimuli, such as activation of pattern recognition receptors (PRRs). PRRs include various Toll-like receptors (TLRs) and scavenger receptors and can be activated by oxidized lipids, modified LDL and saturated fatty acids that are present in atherosclerotic lesions [47]. Activation of PRRs in macrophages can lead to apoptosis induction via the CD36-TLR2 pathway accompanied with NADPH oxidase-mediated oxidative stress. Up-regulation of NADPH oxidase can also stimulate the PERK-CHOP pro-apoptotic signalling. The “two hit” hypothesis therefore suggests that ER stress in macrophages present in atherosclerotic lesions can be induced by low doses of stress agents, such as PRR ligands, which, in turn, induces macrophage apoptosis [47,48]. ER stress-induced macrophage apoptosis is likely to be a key event in transformation of benign lesions to life-threatening vulnerable plaques that can induce thrombus formation [49].

The transition from stable to vulnerable plaque may also be dependent on apoptosis induction in vascular smooth muscle cells (VSMCs), which alters the formation of the protective fibrous cap [50]. ER stress-induced apoptosis in VSMCs is therefore an important event in the disease pathogenesis, although it remains much less studied than apoptosis in macrophages or endothelial cells. CHOP-induced apoptosis can be triggered in VSMCs by a number of ER stress factors, including 7-KC, free cholesterol, homocysteine and others [51,52]. The process is associated with increased oxidative stress, suggesting that treatment with antioxidants may have a protective role [53]. Lipid accumulation in VSMCs is associated with up-regulation of sterol element binding protein-2 (SREBP-2), which also activates ER stress upon treatment with homocysteine [54,55]. In diabetes, which is frequently associated with atherosclerosis, glucosamine accumulation in vascular cells may induce ER stress accompanied by GRP78 up-regulation [56]. Further studies are needed to delineate the role of ER stress signalling in VSMCs in atherosclerosis development.

## 5. Therapeutic Potential of ER Stress Modulators for Treatment of Atherosclerosis

Agents targeting the components of ER stress pathways can have a high therapeutic value for treatment of conditions, in which ER stress and UPR play prominent roles, including atherosclerosis. One of the possible mechanisms of ER stress alleviation is the use of chemical chaperones, such as phenylbutyrate and tauroursodeoxycholic acid (TUDCA). These agents facilitate protein folding and trafficking in a non-selective manner, therefore decreasing the protein load of ER in stress conditions [57]. Phenylbutyric acid (PBA) is currently approved for treatment of urea cycle disorders and is clinically tested for treatment of other diseases associated with protein misfolding [58,59]. In diabetic mice, PBA reduced ER stress and normalized glucose level [60]. A recent study conducted on ApoE knock out atherosclerotic mice demonstrated that treatment with PBA resulted in alleviation of the ER stress and suppressed the up-regulation of CD36, GRP78 and IRE1 phosphorylation in macrophage-rich lesions. CD36 inhibition decreased the ATF6, IRE1, XBP1 and GRP78 signalling induced by modified LDL [61]. It would be interesting to test for possible beneficial effects of PBA in human cardiovascular pathology. TUDCA was also demonstrated to reduce ER stress and attenuate atherosclerotic lesion progression in mice, deficient for LDL receptor [62]. The chemical chaperone also demonstrated protective effects *in vitro* by preventing oxLDL-induced ER stress in murine macrophages transgenic for human APOE4, a genetic risk variant for atherosclerosis [63].

Another path to alleviation of the ER stress is restoration of proteasome function to help reduce the unfolded protein load [64]. Protein kinase A activators, such as isoproterenol and forskolin, employ this mechanism to attenuate ER stress and associated apoptosis [65]. On the other hand, inhibition of protein synthesis via eIF2α signalling can also have protective effects under the ER stress conditions. Salubrinal, which specifically inhibits eIF2α phosphatases [66], could prevent ER stress-induced apoptosis in cardiomyocytes through inhibition of CHOP-mediated pro-apoptotic signalling [67]. However, salubrinal was also demonstrated to induce a severe ER stress and apoptosis in pancreatic β-cells [66], indicative that the response to treatment may be cell type-specific.

Furthermore, inhibition of signalling pathways up-regulated in ER stress can have protective effects [68]. For instance, inhibition of TNF-α with pravastatin or specific antibodies could protect cardiomyocytes from apoptosis [69,70]. Although CHOP is regarded as one of the most exciting potential therapeutic targets for ER stress alleviation in metabolic disorders, only a few pharmacological inhibitors of this pathway have been described so far. CHOP phosphorylation could be prevented by treatment with a p38 mitogen-activated protein kinase (MAPK) SB203580 [71], and JNK inhibitor SP600125 could suppress mechanical stress-induced activation of CHOP [70]. Valproate, which has been approved for treatment of bipolar disorder and epilepsy, induces the expression of the ER chaperone BiP and suppresses induction of CHOP and caspase-12 activation [72]. The drug has demonstrated beneficial effects in atherosclerotic ApoE knock out mice, opening interesting therapeutic opportunities [73].

Several specific inhibitors of ERO1 have been developed. Compounds EN460 and QM295 were demonstrated to induce the adaptive UPR response to alleviate the ER stress [74,75]. Another example of signalling pathways implicated in regulation of ER stress is AMP kinase (AMPK) signalling. AMPK is one of the cellular energy sensors, which performs the switch between anabolic and catabolic pathways. Its inactivation is associated with severe ER stress and atherosclerosis that can be alleviated by the ER stress suppressors, such as tempol and TUDCA [40]. On the other hand, activators of AMPK, such as 5-aminoimidazole-4-carboxyamide-1-β-D-ribofuranoside (AICAR), atorvastatin, A-769662 and PT1 have protective effects on cardiac cells by reducing the ER stress. AMPK agonists are currently used for treatment of obesity and metabolic syndrome, however, clinical implication of these drugs may be broadened [76,77].

Restoration of the ER calcium homeostasis is another promising avenue for alleviation of ER stress-induced apoptosis. Several existing drugs, including verapamil and diltiazem can inhibit calcium efflux from the ER enhancing its protein folding capacity [78,79]. Moreover, mitochondrial calcium homeostasis is linked to that of the ER and its modulation may also have beneficial effects in metabolic disorders [68]. However, more studies are needed to determine the therapeutic potential of calcium regulation in atherosclerosis.

Naturally occurring chemical compounds often have therapeutic properties targeting different metabolic and signalling pathways. A wide spectrum of natural components that have potential for alleviation of the ER stress and apoptosis has recently been reviewed by Pereira *et al.* [80]. Known ER stress-targeting natural components include proteasome inhibitors, such as brefeldin A, tunicamycin, lactacyclin and others, regulators of the ER calcium homeostasis, such as thapsigargin, basiliolide A1 and agelasine B, IRE1/PERK signalling inhibitors, such as resveratrol and withaferin A and other active molecules.

## 6. Conclusions

As the role of the ER stress in atherosclerosis development becomes evident, more potential drug targets for possible therapy improvement are reported. However, it has to be kept in mind that the UPR has important adaptive functions, and the same molecules are often implicated both in adaptive and pathological pathways. Therefore, therapy targeting ER stress and apoptosis in atherosclerosis has to be fine-tuned to avoid severe adverse events. Different cell types involved in the disease pathogenesis, such as macrophages, endothelial and smooth muscle cells can have different responses to the ER stress. Knowledge of the molecular mechanisms of ER stress has accumulated during the last years; however, many questions remain unanswered. Future studies should focus on assessment of novel possible therapeutic targets and on evaluation of safety and efficacy of existing drugs targeting the ER stress pathways in order to evaluate their potential clinical utility for treatment of atherosclerosis.
